# Kinetic modelling of the solid–liquid extraction process of polyphenolic compounds from apple pomace: influence of solvent composition and temperature

**DOI:** 10.1186/s40643-021-00465-4

**Published:** 2021-11-24

**Authors:** Parinaz Hobbi, Oseweuba Valentine Okoro, Christine Delporte, Houman Alimoradi, Daria Podstawczyk, Lei Nie, Katrien V. Bernaerts, Amin Shavandi

**Affiliations:** 1 Université Libre de Bruxelles (ULB), École polytechnique de Bruxelles - BioMatter unit, Avenue F.D. Roosevelt, 50 - CP 165/61, 1050 Brussels, Belgium; 2 Laboratory of Pathophysiological and Nutritional Biochemistry, Université Libre de Bruxelles, Brussels, Belgium; 3 School of Biomedical Sciences, University of Otago, Dunedin, New Zealand; 4 Department of Process Engineering and Technology of Polymer and Carbon Materials, Faculty of Chemistry, Wroclaw University of Science and Technology, Norwida 4/6, 50-373 Wroclaw, Poland; 5 College of Life Sciences, Xinyang Normal University (XYNU), Xinyang, 464000 China; 6 Faculty of Science and Engineering, Brightlands Chemelot Campus, Aachen-Maastricht Institute for Biobased Materials (AMIBM), Maastricht University, Urmonderbaan 22, 6167 RD Geleen, the Netherlands

**Keywords:** Apple pomace, Polyphenolic compounds, First-order kinetic model, Second-order kinetic model, Waste valorization, Value extraction

## Abstract

**Abstract:**

This study aims to assess kinetic modelling of the solid–liquid extraction process of total polyphenolic compounds (TPC) from apple pomace (AP). In this regard, we investigated the effects of temperature and solvent (i.e. water, ethanol, and acetone) on TPC extraction over various periods. The highest TPC yield of 11.1 ± 0.49 mg gallic acid equivalent (GAE)/g db (dry basis) was achieved with a mixture of 65% acetone–35% water (v/v) at 60 °C. The kinetics of the solvent-based TPC extraction processes were assessed via first-order and second-order kinetic models, with an associated investigation of the kinetic parameters and rate constants, saturation concentrations, and activation energies. The second-order kinetic model was sufficient to describe the extraction mechanism of TPC from AP. This study provides an understanding of the mass transfer mechanism involved in the polyphenolic compound extraction process, thus facilitating future large-scale design, optimization, and process control to valorize pomace waste.

**Graphical Abstract:**

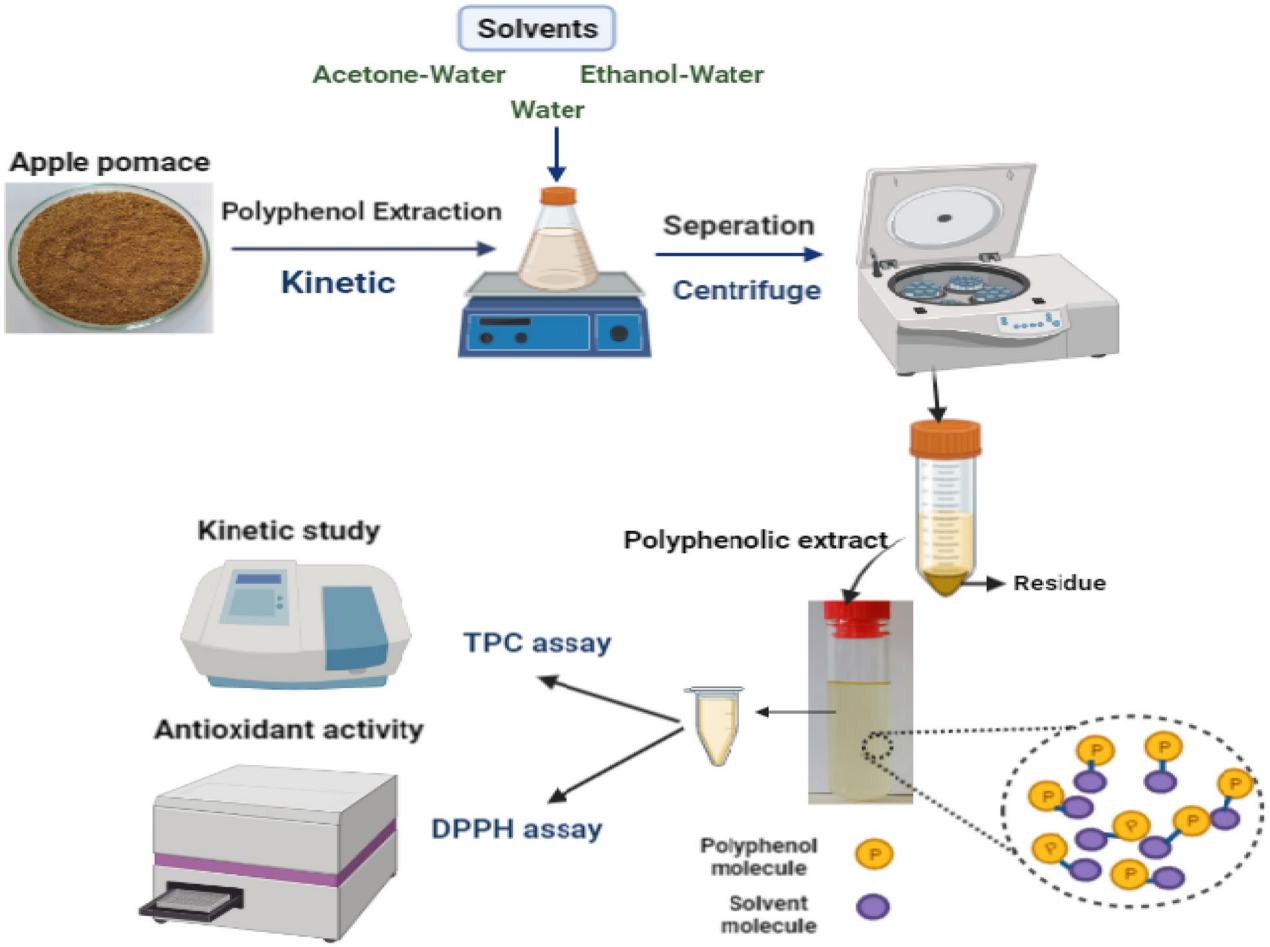

**Supplementary Information:**

The online version contains supplementary material available at 10.1186/s40643-021-00465-4.

## Introduction

Apple pomace (AP) is the main residue obtained from the apple processing industries of wine, jam, juice, cider and constitutes 25–30% of the mass of processed apple (Antonic et al. 2020). About 10 million tonnes of AP are generated globally every year (Alongi et al. 2019). One of the most common approaches for disposing of this by-product is through direct discarding to the soil in a landfill causing environmental pollution (Bhushan et al. 2008). Indeed, once applied to fields, AP, characterized by high moisture content (70–75%) and high biodegradability, encounters microbial decomposition leading to greenhouse gas (GHG) emission (Bhushan et al. 2008; Okoro et al. 2021).

In the context of environmental management and the circular paradigm (Okoro et al. 2017), recycling AP is of great interest due to the presence of various compounds, such as polysaccharides, dietary fibres, minerals, and bioactive polyphenolic compounds which can be utilized for the production of a wide range of high-value compounds (Lyu et al. 2020a). AP is a mixture of skin and flesh (95 wt.%), seeds (2–4wt.%), and stems (1 wt.%) (Perussello et al. 2017), which contain different polyphenolic compounds such as epicatechin, quercetin, phloretin, chlorogenic acid, protocatechuic acid, caffeic acid, p-coumaric acid, ferulic acid, salicylic acid, phloridzin, and 3-hydroxyphloridzin (Antonic et al. 2020; Lyu et al. 2020a). The antioxidant, anti-inflammatory, anti-cancer, and anti-diabetic properties of some of these polyphenolic compounds are well-characterized (Shavandi et al. 2018). Moreover, in recent years, their applications in biomaterial engineering for the stimulation of bone formation and remineralization, fabrication of anti-diabetic wound healing scaffolds have also attracted considerable attention (Shavandi et al. 2018). Given the abundance of AP and complications of current disposal methods, there has been an increasing interest in value extraction from this organic waste stream, particularly as a sustainable source of useful polyphenolic compounds.

Among various extraction methods used for the recovery of polyphenolics from organics such as solid–liquid extraction, microwave-assisted extraction, pressurized liquid extraction, ultrasonic, electric field assisted extraction and supercritical fluid extraction (Perussello et al. 2017), solid–liquid extraction using solvents such as ethanol, methanol, acetone, and their mixtures with water constitute the most widely used extraction techniques (Li et al. 2020). These solvents are characterized by high extraction efficiencies in the recovery of wide ranges of polyphenols with different structures and are cheaper compared to other methods. Moreover, the application of optimization process in soild–liquid extraction provides a suitable extraction rate and yield (Li et al. 2020; Perussello et al. 2017). For instance, a step-wise fractionation method for the extraction of polyphenolic compounds from AP using water at room temperature and subsequent extractions using methanol and then acetone was investigated by Reis et al. (2012). Their study confirmed the efficiency of water and aqueous methanol and acetone solvents for the extraction of water-soluble and organic solvent-soluble polyphenolic compounds with significant antioxidant activity. In the same study, water was suggested as a potential solvent for the extraction of different kinds of polyphenolic compounds from AP which is environmentally friendly and cheap. To maximize the yield of extracted polyphenolic compounds from AP, several studies have been carried out to optimize experimental extraction conditions. For instance, Zardo et al. (2020) utilized acetone, ethanol, and methanol to enhance the extraction of polyphenolic compounds from AP by investigating the effect of particle size, ratio of AP to solvent, and solvent concentration on the total polyphenolic compound (TPC) yields. The use of different solvents may result in the recovery of different types and concentrations of TPC. For instance in the study by Wijngaard and Brunton (2010), the extraction of polyphenolic compounds by acetone and ethanol was optimized and TPC value of 1415 mg GAE/100 dry basis (db) and 1092 mg GAE/100 db was achieved, respectively. The results of this study demonstrated that these two solvents could be preferentially employed when compared with methanol to facilitate polyphenolic extraction. In addition, acetone and ethanol are considered more environmental friendly solvents than methanol (Wijngaard and Brunton 2010).

Mathematical modelling has been considered as an important step in process engineering such as extraction processes due to the fact that the influence of process parameters on the outcome of the process is quickly determined with minimizing the number of needed experiments. Solid–liquid extraction is a heterogeneous, multicomponent process in which the mass transfer mechanism from solid to liquid happens at different rates. Application of kinetic model and analyses of model parameters provides a good realization of the extraction mechanism (Bucic-Kojic et al. 2013). Despite the importance of TPC extraction kinetics in optimization of the methods, this has not been adequately studied so far.

The kinetic modelling of TPC extraction plays a key role in the scaling-up the process for industrial applications. Furthermore, given that temperature and extraction time are considered as the two main parameters that may influence the efficiency of TPC extraction, undertaking kinetic modelling of the extraction process is necessary to aid future process design via reducing energy, time and chemical reagent consumption (Jurinjak Tušek et al. 2016; Shewale and Rathod 2018). In a study (Lazar et al. 2016), the second-order kinetic model was successfully applied to describe the mechanism of the ultrasound-assisted extraction of polyphenols from spruce bark (*Picea abies*), in which ultrasound and temperature provided significant effect on extraction yield and kinetics of the polyphenols extraction. Another study (Qu et al. 2010) also successfully developed a second-order kinetic model for describing the extraction process of polyphenols compounds from pomegranate marc under different extraction parameters, such as temperature, particle size and solid/solvent ratio.

First-order kinetic model was applied for the conventional and ultrasound-assisted extractions of polyphenol from AP and comparing the obtained kinetic parameter (K) showed that both extraction methods followed the same first-order kinetic models (Pingret et al. 2012). However, to the best of our knowledge no more studies have been carried out on kinetic modelling of polyphenol extraction from AP. This study, therefore, is designed to investigate the kinetics of TPC extraction from AP influenced by temperature and solvent composition and determine the best-fitting kinetic model for the extraction process. Given that the first- and second-order kinetic models are commonly used to investigate adsorption, desorption and extraction processes, we applied these models under the non-equilibrium conditions to model the solid–liquid extraction process of polyphenolic compounds from AP. The effect of three environmentally benign solvents, i.e. water, ethanol, and acetone, were assessed in terms of the yield of the TPC extracted and the associated kinetic parameters of extraction rate constants, saturation concentrations, and activation energies.

## Materials and methods

### Materials

Apple pomace containing apple flesh, skin, seeds, and stems were acquired from Materne-Confilux Company (Namur, Belgium). The samples were well mixed to achieve homogeneity. The acquired samples were subsequently dried in an oven to constant mass at a temperature of 60 °C for 24 h. The dried apple sample was ground to a fine powder and sieved using 0.25-mm Endecott mesh. The samples were then stored in airtight Tedlar bags and preserved in a − 18 °C in freezer. Ethanol (Reagent grade VWR Chemicals, Belgium), acetone (Reagent grade VWR Chemicals, Belgium), methanol (Analytical grade, ProLabo, EEC), Folin–Ciocalteu’s phenol reagent (analytical grade, Chemical Lab, Belgium), 2,2-diphenyl-1picrylhydrazyl radical (DPPH) (Analytical grade, Sigma-Aldrich, St Louis, MO, USA), Gallic acid (Analytical grade, Sigma-Aldrich, St Louis, MO, USA) and sodium carbonate (Reagent grade Merck chemical, Darmstadt, Germany) were utilized as chemical inputs.

### Methods

#### Waste apple pomace (WAP) characterization

Proximate analyses to determine the moisture content, fixed carbon content, volatile matter and ash contents of the WAP sample were undertaken according to the standard methods of the ASTM E1756-08 (ASTM 2015), ASTM method D3172-07 (ASTM 2007), ASTM D3175-11 (ASTM 2011), and ASTM D 2017-98 (ASTM 1998), respectively. The elemental analysis was undertaken using an elemental analyzer (LECO TruSpec CHN, Saint Joseph, Michigan, USA) to determine the carbon, hydrogen, nitrogen, and sulphur contents of the WAP sample. The elemental oxygen content was determined by subtracting the fractional ash, carbon, hydrogen, nitrogen, and sulphur contents from unity (Okoro et al. 2018). The lipid content was determined using the Soxhlet method (Kim et al. 2012). The protein content was determined according to the AOCS official method Ba4e-93 (AOAC 1998). The lignin content was determined using the Klason method (Carrier et al. 2011). The total carbohydrate content was determined by the difference (Okoro et al. 2018).

#### Extraction procedures of polyphenolic compounds

Solid–liquid extractions using three different solvents of water-only (designated as 100 WA henceforth), 50% ethanol–50% water v/v (designated as 50 ETH henceforth), and 65% acetone–35% water v/v (designated as 65 ACE henceforth) were carried out for the recovery of polyphenolic compounds present in dry AP sample. To this end, the extraction operating parameters were selected based on previous optimization studies for enhanced TPC extraction (Candrawinata et al. 2014; Ibrahim et al. 2019; Zardo et al. 2020). Optimal TPC extraction with 100 WA was attained at solid (dry apple pomace) mass-to-solvent volume ratio of 1:20 g/mL (Candrawinata et al. 2014). The optimal TPC extraction using ethanol has been achieved using 50 ETH and a solid-to-solvent ratio of 1:80 g/mL (Zardo et al. 2020). When 65 ACE was employed, the solid-to-solvent ratio of 1:100 g/mL was imposed (Ibrahim et al. 2019). The experiments were undertaken in a 50 mL laboratory flask with continuous stirring at 200 rpm. For the kinetic studies, when 100 WA was used for extraction, the experiments were run at 85 °C, 60 °C, and 40 °C. For extraction with 50 ETH or 65 ACE, the experiments were carried out at 60 °C, 40 °C, and 20 °C. In all mentioned experimental conditions, extraction times were assessed at 5 min, 10 min, 15 min, 20 min, 25 min, 30 min, and 40 min. After the extraction, the samples were centrifuged at 6000*g* for 10 min for extraction with ethanol and acetone, and 9000*g* for 10 min for water extraction. Finally, the supernatant was carefully collected for TPC determination.

#### Determination of total polyphenolic content (TPC)

The TPC of the extracts was determined using the Folin–Ciocalteu colorimetric method as described in the literature (Li et al. 2020). Briefly, 50 μL of the extract was mixed with 250 μL Folin–Ciocalteu reagent (2 M) and 3 mL of distilled water and vortexed for 10 s. Then, 1 mL of 15% (w/v) Na_2_CO_3_ solution was added, and subsequently, the volume of the mixture was brought up to 5 mL by adding 700 μL of distilled water, vortexed for another 10 s and incubated at 20 °C in dark for 1 h. Finally, the absorbance of the resulting solution was measured using UV–visible spectrophotometer (PerkinElmer Lambda 25, MA, USA) at 765 nm. The TPC was expressed as milligrammes of gallic acid equivalents (determined by a standard curve) per gramme of dry weight basis of AP (mg GAE/g db) as follows:

1 $$\text{TPC} = \frac{C \times V}{m},$$

where *C* denotes the sample concentration (mg/mL) obtained from the standard curve, *V* is the volume of the solvent used for the extraction, and *m* represents the weight (g) of the dried AP sample used for the extraction.

#### Kinetic modelling for the extraction methods

In this study, first-order and second-order kinetic models were employed in modelling the extraction of bioactive polyphenolic compounds from AP by considering solid–liquid extractions using three different solvents of 100 WA, 50 ETH, and 65 ACE. Briefly, the first-order extraction kinetic model as proposed by Harouna-Oumarou et al. (2007) has been assessed such that the rate of leaching (*r*_*e*_) is proportional to a driving force (*C*_*s*_*-C*_*t*_) and the first-order rate equation is correlated with the idea of a linear driving force as shown in Eq. (2):

2 $$r_{e} = \frac{{\text{d}C_{t} }}{\text{d}t} = k\left( {C_{s} - C_{t} } \right),$$

where *C*_*t*_ (mg GAE/g db) is the extraction capacity (concentration of TPC) at a given extraction time *t*, *C*_*s*_ (mg GAE/g db) is the concentration of TPC at saturation point and *k* is the first-order extraction rate coefficient (min^−1^).

A linear equation (Eq. 3) is obtained by the integration of Eq. (2) at the boundary condition*s* of *C*_*t*_ = 0 at *t* = 0 and *C*_*t*_ = *C*_*t*_ at *t* = *t,* such that plotting ln values against *t* provides the slope that can be used in the determination of first-order extraction rate constant:

3 $${\text{ln}}\left[ {\frac{{C_{s} }}{{C_{s} - C_{t} }}} \right] = kt.$$

In such solid–liquid extractions, the Arrhenius-type equation was proposed to investigate the relation of extraction rate to temperature and so the temperature dependence of the extraction kinetics (Balyan and Sarkar 2016).

Therefore, further determination of the kinetic parameters was based on the Arrhenius-type equation as shown in Eqs. (4) and (5) (Shewale and Rathod 2018). The kinetic parameters (*A*_*e*_ and *E*_*a*_) are calculated by plotting ln *k*, against 1/*T* (Eq. 4) and the slope and the intercept give the *E*_*a*_ and the *A*_*e*_, respectively:

4 $$k = A_{e} e^{{ - \left[ {\frac{{E_{a} }}{RT}} \right]}} ,$$

5 $$\ln k = \ln A_{e} - \frac{{E_{a} }}{RT}.$$

In Eqs. (4) and (5), *k, A*_*e*_*, E*_*a*_*, R,* and *T* represent the approximate overall rate constant, in min^−1^; pre-exponential constant (Arrhenius constant), in min^−1^; activation energy, in kJ/kmol; universal gas constant, specified as 8.314 kJ/kmol.K and temperature in K, respectively.

The second-order extraction kinetics is, however, modelled using Eq. (6) (Harouna-Oumarou et al. 2007):

6 $$r_{e} = \frac{{\text{d}C_{t} }}{\text{d}t} = k\left( {C_{s} - C_{t} } \right)^{2} .$$

Such that the integration of Eq. (6), using the boundary condition*s C*_*t*_ = 0 at *t* = 0 and *C*_*t*_ = *C*_*t*_ at *t* = *t* is as follows:

7 $$\frac{1}{{C_{s} - C_{t} }} - \frac{1}{{C_{s} }} = kt,$$

or

8 $$C_{t} = \frac{{C_{s}^{2} kt}}{{1 + C_{s} kt}}.$$

Equation (8) was then rearranged in a linearized form to give Eqs. (9) and (10) as follows:

9 $$\frac{t}{{C_{t} }} = \frac{t}{{C_{s} }} + \frac{1}{{C_{s}^{2} k}},$$

10 $$\frac{t}{{C_{t} }} = \frac{t}{{C_{s} }} + \frac{1}{m},$$

where *m* denotes the initial extraction rate coefficient and is equal to *kC*_*s*_^*2*^*.*

The second-order extraction rate coefficient is calculated from the intercept obtained through plotting *t/C*_*t*_ against *t* using Eq. (10).

The values of the other kinetic parameters (*A*_*e*_, *E*_*a*_) were determined using Eqs. (4) and (5), where *k* and *A*_*e*_ have units of g/(mg min) for the second-order extraction process.

All experiments were conducted in triplicates and data were subsequently analysed.

#### Determination of the antioxidant activity of apple pomace extracts based on DPPH inhibition activity

The radical scavenging capacity of TPC extracts of AP against 2,2-diphenyl-1 picrylhydrazyl radical (DPPH) was conducted based on the method described in de Torre et al. (2019). The sample extracts of 100 WA, 50 ETH, and 65 ACE containing the highest TPC concentration were selected as representative extracts for the DPPH determination experiments. 150 µL of each extract was mixed with 150 µL of DPPH solution in methanol (0.04 mg/mL) in a 96-well plate. After incubation in dark for 40 min, the absorbance of the sample at 517 nm was recorded using a UV–Vis Spectrophotometer (Microplate spectrophotometer, Epoch-BioTek, Winooski, USA), and the percentage of DPPH inhibition activity of the sample extract was calculated as follows:

11 $$\% {\text{Inhibition}} = 1 - \frac{{{\text{Abs}}_{s} - {\text{Abs}}_{b} }}{{{\text{Abs}}_{c} - {\text{Abs}}_{b} }} \times 100,$$

where Abs_*s*_ denotes the absorbance of the sample (sample extract + DPPH solution), Abs_*b*_ denotes the absorbance of the sample blank (sample extract + methanol), and *Abs*_*c*_ denotes the absorbance of the control (extraction solvent + DPPH solution).

## Results and discussion

### Waste apple pomace characterization

The physicochemical characterization of AP is displayed in Table 1. Based on the elemental analysis, the greatest elemental amount corresponds to carbon with a 46.1% w/w, followed by oxygen with 46.0% w/w and hydrogen with 6.87% w/w. Nitrogen and sulphur with the values of 0.95 and 0.07% w/w constituted the lowest amounts in AP. Our results are in accordance with those found in previous studies with ranges obtained at 44.6–48.8% w/w for carbon, 37.4–45.7% w/w for oxygen, 6.18–6.65% w/w for hydrogen, 0.42–1.70% w/w for nitrogen (Gowman et al. 2019). The moisture content was detected as 67.3% which has been reported to range from 40 -82.7% (Okoro and Shavandi, 2021). Moreover, there was low ash content (1.30% w/w) and high volatile content (92.4%) which are within the range of 0.50–4.29% (Antonic et al. 2020) and comparable to 81.3% (Guerrero et al. 2014) for ash and volatile contents, of typical lignocellulosic biomass. The mean contents of carbohydrate, protein, and lipid were determined at 71.9% w/w, 5.94% w/w, and 1.29% w/w, respectively, and was within the ranges of 48–84% w/w for carbohydrate (Bhushan et al. 2008; Lyu et al. 2020b), 1.2–6.91% w/w for protein and 0.26–8.49% w/w for lipid contents (Antonic et al. 2020) reported in the literature. The lignin content of 19.5% w/w was also within the range expected in AP 15.3–23.5% w/w (Vidović et al. 2020).

**Table 1:** Characterization results for waste apple pomace

WAP characterization	Measured value
Moisture content (% w/w, wet WAP basis)	67.3 ± 1.06
Lipid content (% w/w, dry WAP basis)	1.29 ± 0.52
Carbohydrate content (% w/w, dry WAP basis)	71.9 ± 1.30
Protein content (% w/w, dry WAP basis)	5.94 ± 0.20
Lignin content (% w/w, dry WAP basis)	19.5 ± 1.18
Ash content (% w/w, dry WAP basis)	1.30 ± 0.00
Volatiles (% w/w, dry WAP basis)	92.4 ± 0.00
Fixed carbon(% w/w, dry WAP basis)	6.34 ± 0.00
Carbon content (% w/w, dry WAP basis)	46.1 ± 0.64
Hydrogen content (% w/w, dry WAP basis)	6.87 ± 0.11
Nitrogen content (% w/w, dry WAP basis)	0.95 ± 0.03
Oxygen content (% w/w, dry WAP basis)	46.0 ± 0.65
Sulphur content (% w/w, dry WAP basis)	0.07 ± 0.01

### Kinetics of water-only extraction of total polyphenolic compounds from waste apple pomace

Experimental results for the extraction kinetics of the TPC from AP at different temperatures (40 °C, 60 °C, and 85 °C) with water-only are depicted in Table 2. A rapid increase in extracted TPC was observed at the beginning of the process (~ within 5 min). Then the gradual and slow increase trend was distinguished in the further progress of the extraction process and continued till the peak point was reached where a maximum of TPC was extracted. This observation can be explained based on Fick’s law. According to Fick’s law, at the beginning of an extraction process, high concentration gradient between the solid phase (AP) and liquid phase (solvent) results in high diffusion of polyphenolic compounds into the solvent. As the extraction continues, the concentration gradient gets smaller; thereby increasing the extraction yield until the peak point is attained (Harouna-Oumarou et al. 2007). Notably, Table 2 also shows that the extracted TPC was reduced beyond the peak point in all conditions. This observation may be attributed to the oxidation and decomposition of TPC leading to thermal degradation of polyphenolic compounds with sustained heating and long extraction time (Narayana Namasivayam et al. 2018; Perussello et al. 2017). This statement is consistent with literature since polyphenol stability, is recognized as a challenge for the food industry and is sensitive to temperature, pH and even light exposure (Diaconeasa 2018). Therefore, in assessing the kinetics of TPC extraction, the values beyond the peak values have not been considered in the modelling. Indeed, they do not reflect the extraction kinetics of TPC but are rather a representation of the thermal degradation rates of the extracted TPC. Table 2 also shows that the highest TPC value at 60 °C is 5.10 ± 0.06 mg GAE/g db in 30 min, while at temperatures of 85 °C and 40 °C, the highest TPC values of 4.45 ± 0.18 mg GAE/g db and 3.67 ± 0.08 mg GAE/g db were achieved, respectively. The results indicate that the influence of changes in temperature constitutes an important extraction parameter that influences the recovery of TPC, as expected. The influence of increasing temperature on the extraction efficiency of TPC could be due to the increased solubility of polyphenolic compounds in the solvent and their enhanced diffusion rate and mass transfer from the solid matrix to the solvent (Shewale and Rathod, 2018). However, the maximum TPC yield of 4.45 ± 0.18 mg GAE/g db obtained at 85 °C was lower than the TPC value of 5.09 ± 0.06 mg GAE/g db at 60 °C. This could be due to the thermal degradation of heat-sensitive polyphenolic compounds (Perussello et al. 2017), discussed above.

**Table 2:** Total polyphenolic compound (TPC) extraction from apple pomace (AP) with water-only at solid/solvent ratio 1:20 g/mL, under continuous magnetic stirring at 200 rpm

Temperature (°C)	Time (min)	TPC (mg GAE/g db)
85	0	0
	5	3.02 ± 0.12
	10	4.14 ± 0.02
	15	4.27 ± 0.11
	25	4.36 ± 0.18
	30	4.45 ± 0.18
	40	3.51 ± 0.14
60	0	0
	5	4.15 ± 0.15
	10	4.29 ± 0.13
	15	4.36 ± 0.09
	25	4.93 ± 0.14
	30	5.09 ± 0.06
	40	3.33 ± 0.30
40	0	0
	5	2.28 ± 0.04
	10	2.67 ± 0.12
	15	3.07 ± 0.06
	25	3.66 ± 0.08
	30	3.33 ± 0.08

#### First-order kinetic model for water-only extraction of TPC from waste apple pomace

**Fig. 1:**
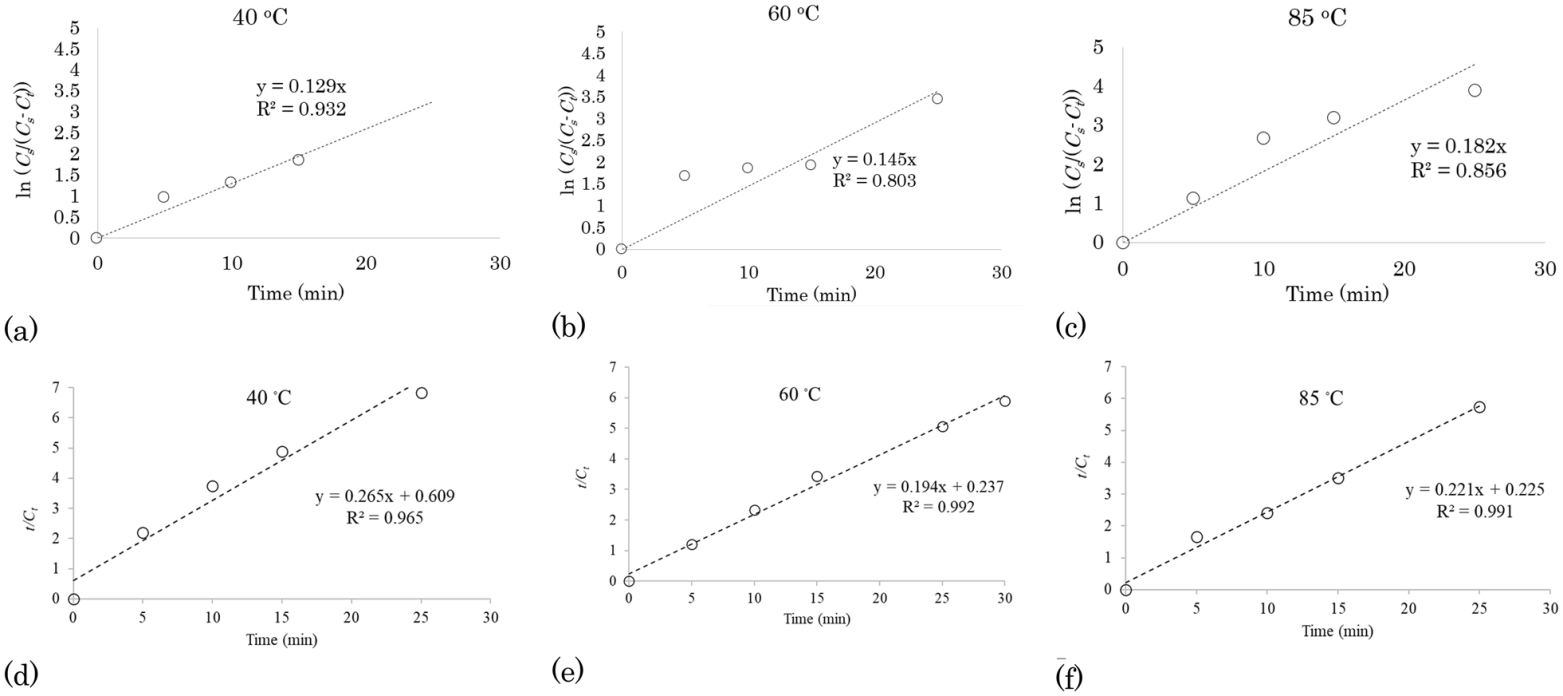
First-order kinetic models (**a**–**c**) and second-order kinetic models (**d**–**f**) of extraction of total polyphenolic compounds from apple pomace with water-only at different temperatures

For the first-order kinetic model, the experimental data of the extraction kinetics (Table 2) were plotted as ln (*C*_*s*_/(*C*_*s*_*-C*_*t*_)) versus *t.* The resulting plot was used to determine the values of first-order rate constant (*k*) and coefficient of determination (R^2^) (Fig. 1a–c). The plot shows that the extraction of TPC of AP can be represented by a linear form of the first-order model. The *k* value increased with increasing temperature and obtained values are 0.129 min^−1^, 0.145 min^−1^, and 0.182 min^−1^ at reaction temperatures of 40 °C, 60 °C, and 85 °C, respectively. This observation shows that a positive correlation exists between the extraction rate and the temperature, thus reinforcing the importance of the elevated temperatures on the extraction rate of TPC with 100 WA solvent. The mean *R*^2^ value of the extraction kinetics models obtained for the different temperatures (Fig. 1a–c) was shown to be 0.864 (i.e. from 0.803, 0.856 and 0.932).

#### Second-order kinetic model for water-only extraction of TPC from waste apple pomace

The second-order kinetic model was also assessed to determine its sufficiency in describing the kinetics of TPC extraction from AP. Following Eqs. 9 and 10, plots of *t/C*_*t*_ versus *t* were generated and the values of the second-order rate constant (*k*) and coefficient of determination (*R*^2^), subsequently determined (Figs. 1d–f). The values of the *k* were determined at 0.123 g/(mg min), 0.163 g/(mg min), and 0.224 g/(mg min) at 40 °C, 60 °C, and 85 °C, respectively, showing its increasing trend with a temperature rise. As shown in Fig. 1d–f, comparatively higher *R*^2^ values (an average value of 0.983) were observed for the second-order kinetic model in comparison to the first-order kinetic fit. This observation shows that the second-order kinetics is a more suitable model for describing the kinetic extraction process of TPC with water-only, which can be concluded that more than one mechanism contributes to the extraction process (Harouna-Oumarou et al. 2007).

### Kinetics of ethanol–water extraction of total polyphenolic compounds from waste apple pomace

Experimental results for the extraction of TPC from AP at different temperatures (20 °C, 40 °C, and 60 °C) using 50 ETH are depicted in Table 3. Similar to TPC extraction with water-only, a rapid increase of TPC extraction was observed during the initial 5 min which can be attributed to the rapid diffusion of ethanol–water-soluble TPC into the solvent solution (Cavdarova and Makris 2014). The highest TPC of 9.19 ± 0.36 mg GAE/g db was achieved at 60 °C in 20 min of extraction compared to TPCs of 8.05 ± 0.32 and 7.06 ± 0.47 mg GAE/g db at 40 °C and 20 °C, respectively, thus highlighting the favourable role of (increasing) temperature on TPC extraction. The synergistic effect of the water and alcohol on the TPC extraction efficiency may explain the high TPC achieved. This is because the water and ethanol mixture may increase the contact surface of AP particles and ethanol by weakening the bonds between polyphenolics–protein and polyphenolics–cellulose, thus enhancing the extraction efficiency (Rodriguez De Luna et al. 2020).

**Table 3:** Total polyphenolic compound (TPC) from apple pomace with 50 ETH at solid/solvent ratio of 1:80 g/mL, under continuous magnetic stirring at 200 rpm

Temperature (˚C)	Time (min)	TPC (mg GAE/g db)
60	0	0
	5	8.54 ± 0.20
	10	8.72 ± 0.41
	15	8.85 ± 0.12
	20	9.19 ± 0.36
	25	9.06 ± 0.44
40	0	0
	5	7.24 ± 0.34
	10	7.58 ± 0.12
	15	7.68 ± 0.12
	20	8.05 ± 0.32
	25	7.76 ± 0.35
20	0	0
	5	6.02 ± 0.45
	10	6.38 ± 0.27
	15	6.46 ± 0.36
	20	7.06 ± 0.47
	25	6.77 ± 0.21

#### First-order kinetic model for ethanol–water extraction of TPC from waste apple pomace

**Fig. 2:**
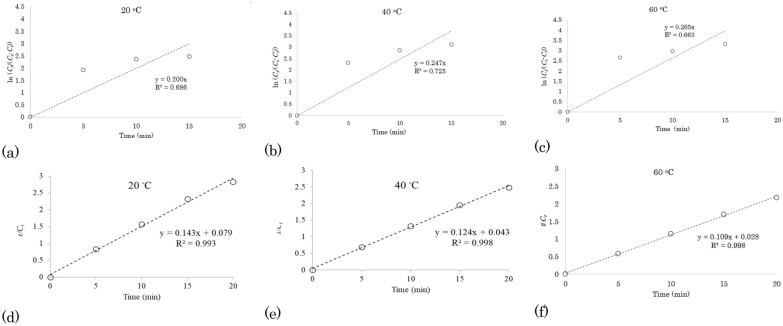
First-order kinetic models (**a**–**c**) and second-order kinetic models (**d**–**f**) of extraction of total polyphenolic compounds from waste apple pomace using 50% ethanol–50% water v/v at different temperatures

The experimental data of the extraction kinetics (Table 3) were processed and by plotting ln (*C*_*s*_/(*C*_*s*_-*C*_*t*_)) versus *t*, the values of rate constant (*k*) and coefficient of determination (R^2^) were obtained (Fig. 2a–c). The *k* values 0.200 min^−1^, 0.247 min^−1^, and 0.265 min^−1^ were obtained at reaction temperatures of 20 °C, 40 °C, and 60 °C, respectively, which shows that raising the temperature could enhance the rate of extraction of TPC. However, relatively low *R*^2^ values ranging from 0.663 to 0.725 (mean 0.691) were achieved which can be concluded that the first-order kinetic model cannot well describe the extraction of TPC of AP with 50 ETH.

#### Second-order kinetic model for ethanol–water extraction of TPC from waste apple pomace

The second-order kinetic model was employed to investigate the release kinetics of TPC extraction from AP by determination of the values of rate constant (*k*) and coefficient of determination (*R*^2^) (Fig. 2d–f). The best fits were acquired when the second-order kinetic model was applied compared with the first-order kinetic model and *k* values were determined to be 0.254 g/(mg min), 0.359 g/(mg min) and 0.419 g/(mg min) at reaction temperatures of 20 °C, 40 °C and 60 °C. Figure 2d–f also shows higher *R*^2^ values ranging from 0.993 to 0.998 (i.e. mean 0.996), which indicates that the second-order kinetic model can be used to study the extraction of TPC from AP with 50 ETH in which temperature has a significant influence on extraction rate.

### Kinetics of acetone–water extraction of total polyphenolic compounds from waste apple pomace

Experimental results for the extraction kinetics of TPC from AP at different temperatures (20 °C, 40 °C, and 60 °C) in 65 ACE are shown in Table 4. Table 4 also shows a rapid increase in the concentration of TPC extraction during the initial 5 min of the reaction followed by a gradual rise. The highest yield of TPC (11.1 ± 0.49 mg GAE/g db) was achieved at the temperature of 60 °C after 30 min of the extraction. At the temperatures of 40 °C and 20 °C, TPC values of 9.37 ± 0.46 and 8.98 ± 0.71 mg GAE/g db were measured, respectively. These observations highlight the influence of temperature on the extraction process. The high efficiency of acetone–water solvent for TPC extraction from AP is attributed to its potential for facilitating the extraction of non-polar and polar TPCs (Mokrani and Madani 2016).

**Table 4:** Total polyphenolic compound (TPC) from apple pomace with 65% aqueous acetone–35% water v/v at solid/solvent ratio of 1:100 g/mL, under continuous magnetic stirring at 200 rpm

Temperature (°C)	Time (min)	TPC (mg GAE/g db)
60	0	0
	5	8.85 ± 0.35
	10	9.05 ± 0.26
	15	9.57 ± 0.44
	20	10.6 ± 0.43
	30	11.1 ± 0.49
	40	10.3 ± 0.50
40	0	0
	5	7.59 ± 0.35
	10	8.11 ± 0.37
	15	8.37 ± 0.35
	20	8.76 ± 0.20
	30	9.37 ± 0.46
	40	8.63 ± 0.20
20	0	0
	5	7.49 ± 0.29
	10	8.07 ± 0.64
	15	8.27 ± 0.10
	20	8.33 ± 0.46
	30	8.98 ± 0.71
	40	8.76 ± 0.35

#### First-order kinetic model for acetone–water extraction of TPC from waste apple pomace

**Fig. 3:**
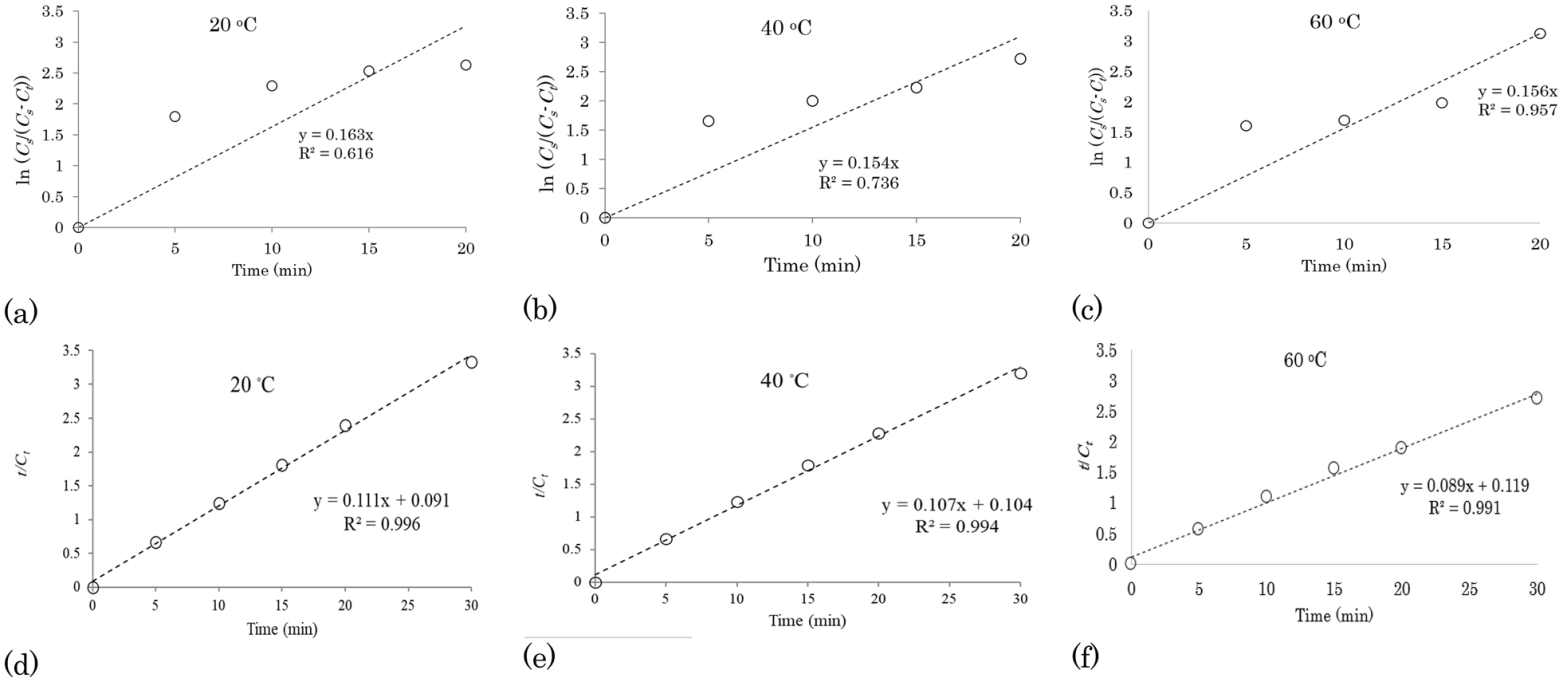
First-order kinetic models (**a**–**c**) and second-order kinetic models (**d**–**f**) of extraction of total polyphenolic compounds from apple pomace with 65% acetone–35% water v/v at different temperatures

First-order kinetic models were achieved based on the experimental data of the extraction kinetics (Table 4). The first-order rate constant (*k*) and coefficient of determination (*R*^2^) were obtained by plotting ln (*C*_*s*_/(*C*_*s*_*- C*_*t*_)) versus *t* (Fig. 3a–c). The extraction of 65 ACE soluble TPC of AP can be represented in the linear form according to the first-order model. The *k* values decreased from 0.163 min^−1^ to 0.154 min^−1^, when the temperature was increased from 20 to 40 °C (Fig. 3d–f); however, at 60 °C, the *k* value (0.156 min^−1^) was comparable to *k* value at 40 °C (0.154 min^−1^). This observation may indicate that there is no positive correlation between the *k* values and the temperatures. Therefore, it can be concluded the rate of TPC extraction from AP using 65 ACE is higher (0.163 min^−1^) at low temperatures (i.e. 20 °C) than the rate of TPC extraction at higher temperatures (0.154 min^−1^ at 40 °C). The efficiency of acetone extraction at low temperatures has been reported in the literature (Zardo et al. 2020). For instance in the research by Mokrani and Madani (2016), extraction of TPC from peach *(Prunus persica L.)* fruit with 60% acetone at 25 °C for 180 min gave the highest TPC of 363 GAE/100 g compared to applying temperatures of higher than 25 °C. We assert that the low temperature sufficiency of acetone to extract high concentrations of polyphenols is due to the ability of acetone to extract polar and non-polar polyphenols since it is comparatively less polar than ethanol and water, with higher temperatures potentially leading to a degradation of polyphenols extracted.

#### Second-order kinetic model for acetone–water extraction of TPC from waste apple pomace

The second-order kinetic models were employed to investigate the release kinetics of TPC from AP by determination of the values of rate constant (*k*) and coefficient of determination (*R*^2^) (Fig. 3d–f). The best fits were acquired when the second-order kinetic model was applied compared with the first-order kinetic model and *k* values were determined 0.158 g/(mg min), 0.109 g/(mg min), and 0.068 g/(mg min) at reaction temperatures of 20 °C, 40 °C, and 60 °C with higher *R*^2^ values (> 0.99), indicating that the second-order kinetic model can be used to study the extraction of TPC from AP with 65 ACE. However, unlike the extraction with 100 WA and 50 ETH, *k* values exhibited a declining trend in response to an increasing temperature which could be confirmed the high efficiency of acetone in the extraction of TPC at low temperatures. Following our result, a study by (Makris and Kefalas 2015), demonstrated that *k* values of the second-order kinetic model of polyphenol extraction from onion *(Allium cepa)* solid waste using the acidified water–ethanol mixture, decreased with increasing temperature from 20 to 40 °C.

### Kinetic parameter determination

The associated kinetic parameters were determined using the Arrhenius-type equation as discussed in "Kinetic modelling for the extraction methods" section above are discussed in the subsequent subsections.

#### Water-only extraction of TPC from waste apple pomace

**Fig. 4:**
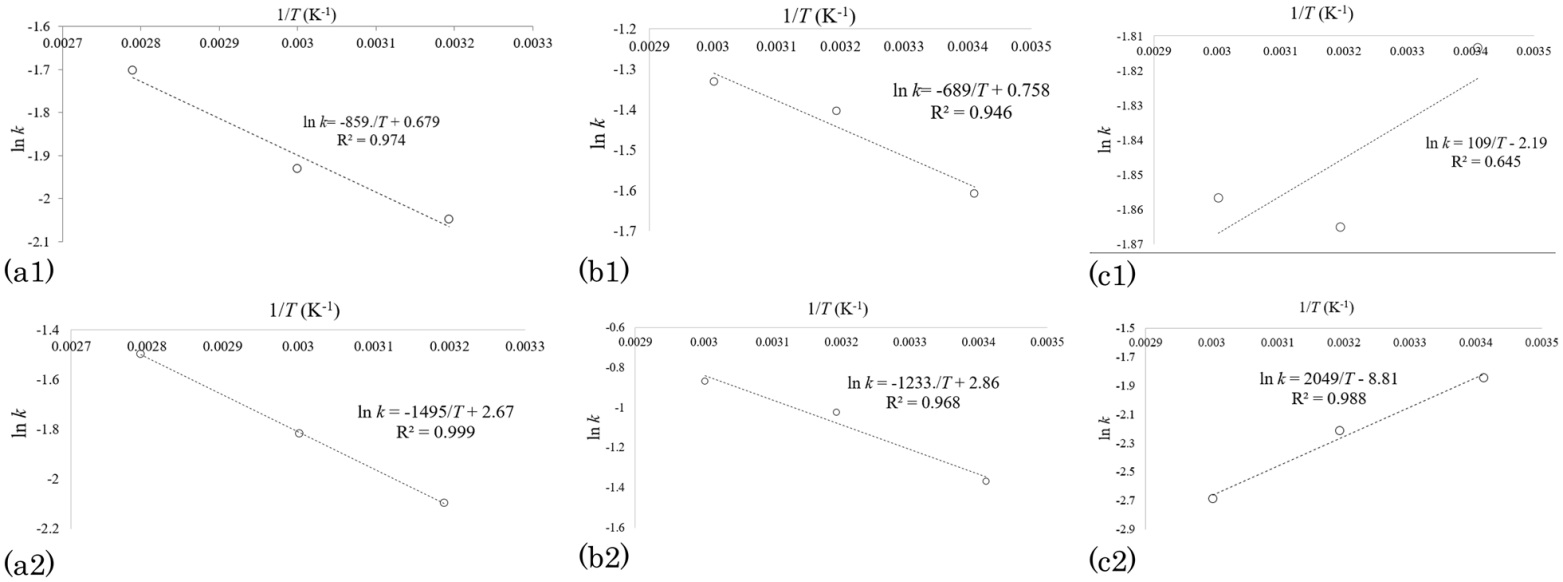
Arrhenius plot obtained from first-order kinetic parameters (**a1**) and from second-order kinetic model parameters (**a2**) for the extraction of total polyphenolic compounds from apple pomace with water-only. Arrhenius plot obtained from first-order kinetic parameters (**b1**) and from second-order kinetic model parameters (**b2**) for the extraction of total polyphenolic compounds from apple pomace with 50% ethanol–50% water v/v. Arrhenius plot obtained from first-order kinetic parameters (**c1**) and from second-order kinetic model parameters (**c2**) for the extraction of total polyphenolic compounds from apple pomace with 65% acetone–35% water v/v

To obtain the kinetic parameters, for the first-order kinetic model case, ln *k* was plotted against 1*/T* (Fig. 4a1), the *E*_*a*_ value for the extraction process was determined to be 7.14 kJ/mol based on the fitted line characterized by the *R*^2^ value of ~ 0.974. The pre-exponential factor, *A*_*e*_, was also determined to be 1.92 min^−1^. The value of *E*_*a*_ in extraction processes depends on different factors, such as the type of solvent, the target compounds, material matrix, and the pre-treatment of the sample (Gonzalez-Centeno et al. 2015). Notably, the classification proposed for the *E*_*a*_ values could be employed in identifying the mechanism that controls the extraction process. Thus, if *E*_*a*_ > 40 kJ/mol, the extraction is managed by solubilization mechanism, if *E*_*a*_ < 20 the extraction is controlled by diffusion mechanism, and if 20 < *E*_*a*_ < 40, the extraction process is governed by a combination of diffusion and solubilization mechanisms (Gonzalez-Centeno et al. 2015). So, considering that the value of *E*_*a*_ is 7.14 kJ/mol, we concluded that the extraction of TPC from AP with water is controlled by a diffusion mechanism. Similarly for the second-order case, using the Arrhenius equation the plot of Fig. 4(a2) was generated. In Fig. 4(a2), the *E*_*a*_ value and pre-exponential factor (*A*_*e*_) were determined to be 12.4 kJ/mol and 14.5 g/(mg min), respectively, with an *R*^2^ value of ~ 0.999. Notably, the *E*_*a*_ value of 12.4 kJ/mol also suggests that the extraction of TPC from AP using water-only is controlled by a diffusion mechanism.

#### Ethanol–water extraction of TPC from waste apple pomace

Using the Arrhenius equation, Fig. 4(b1 and b2) was generated. In Fig. 4(b1), the *E*_*a*_ value and pre-exponential factor for the extraction process were determined to be 5.72 kJ/mol and 2.13 min^−1^, respectively, based on the fitted line characterized by the *R*^2^ value of ~ 0.946. In Fig. 4(b2), the *E*_*a*_ value and pre-exponential factor for the extraction process were determined to be 10.2 kJ/mol (i.e. a diffusion mechanism) and 17.5 g/(mg min), respectively, with the ‘line of best fit’ having an *R*^2^ of ~ 0.968, which is indicative of high levels of data correlation. Regarding the results of the activation energy obtained from the second-order kinetics of 100 WA and 50 ETH extraction, it can be concluded that the extraction of TPC with 50 ETH requires a lower amount of energy which makes this mixture more efficient than water in TPC extraction of AP.

#### Acetone–water extraction of TPC from waste apple pomace

Using the Arrhenius equation and plotting ln *k* obtained from the first-order kinetic models against 1*/T* (Fig. 4c1), considering the low value of *R*^2^ (0.645) and the ascending trend of the fitted line which showed the opposite trend obtained for 100 WA and 50 ETH extractions. The low *R*^2^ value of 0.645 suggests that the kinetic parameters of the extraction using 65 ACE cannot be determined using the Arrhenius equation since the lower temperatures tend to favour TPC extraction (i.e. higher *k* values) as discussed above. For the second-order case (Fig. 4(c2)), the Arrhenius equation was employed to determine the activation energy and pre-exponential factor of the extraction process based on the data obtained for second-order kinetic models. Considering the Arrhenius plot, the high value of *R*^2^ (0.988) was achieved; however, the opposite trend of the fitted line was observed and *E*_*a*_ and *A*_*e*_ values were achieved − 17.0 kJ/mol and 0.00 g/(mg min), respectively, indicating that the extraction with 65 ACE does not obey the Arrhenius equation. This reinforces the assertion that increasing temperatures does not translate to higher TPC when the solvent of 65 ACE is used.

### Overview of the kinetic parameters of the second-order model

Regarding the obtained kinetic data, the second-order kinetic model could adequately describe the extraction of TPC from AP for all the solvents as illustrated by the high coefficient of determination (*R*^2^ > 0.96) values in Table 5. The results showed that temperature is an important factor that influences TPC extraction from AP and has a significant effect on kinetic parameters. Regarding the results of *k* values, higher *k* values were obtained as the extraction temperature increased when 100 WA and 50 ETH solvents were used; however, this trend was not observed when extractions were undertaken using 65 ACE. This result indicates that better extraction efficiency with higher *k* values can be achieved in lower temperatures (< 20 °C) with 65 ACE. Table 5 also shows that the Arrhenius kinetic parameter of *E*_*a*_ ranges from 10.2 kJ/mol to 12.4 kJ/mol for 50 ETH and 100 WA, respectively, confirming the contribution of diffusion mechanism for the extraction of TPC from AP with these solvents. Notably, the values of *E*_*a*_ and *A*_*e*_ were obtained as − 17.0 kJ/mol and 0.00 g/(mg min), respectively, in extraction with 65 ACE. This observation indicates that the Arrhenius equation cannot be applied to determine the kinetic parameters in extraction with 65 ACE because the Arrhenius-based correlation between temperatures and *k* values was not observed. Therefore, the kinetic parameters describing total polyphenolic compound extraction from AP using 65 ACE were not reported in Table 5. For completeness, the statistical significance of the effects of the process parameters of temperature and time on TPC extractions were also assessed via ANOVA investigations in Minitab V17.1.0 (Matlab Inc. USA). The data is presented in the Additional file 1. The parameters were determined to present statistically significant effects on TPC extractions for all solvents.

**Table 5:** Kinetic parameters obtained from second-order model

Solvent	*T* (°C)	*Cs* (mg GAE/g db)	*k* (g/(mg min))	*R*^2^	*E*_*a*_ (kJ/mol)	*A*_*e*_ (g/(mg min))	*R*^2^
100 WA	40	3.77	0.123	0.965	12.4	14.5	0.999
	60	5.15	0.163	0.992			
	85	4.58	0.224	0.991			
50 ETH	20	6.99	0.254	0.993	10.2	17.5	0.968
	40	8.06	0.359	0.998			
	60	9.16	0.419	0.998			
65 ACE	20	9.01	0.158	0.996	–	–	0.988
	40	9.34	0.109	0.994			
	60	11.2	0.068	0.990			

## Determination of the antioxidant activity of waste apple pomace extracts based on DPPH

The DPPH inhibition of the polyphenolic compounds extracted using 100% water, 50% ethanol–water, and 65% acetone–water was determined. The extracts with the highest amount of TPC based on the kinetic results were then assessed. The extract of 65 ACE presented the highest antioxidant activity which can be explained by the higher amounts of TPC obtained with this solvent. The percentage of the DPPH radical scavenging activity was in the order of acetone–water > ethanol–water > water extraction with values of 95.2 ± 3.34, 92.5 ± 1.36, and 71.0 ± 4.66%, respectively. This study also confirms that the antioxidant activity of the extracts correlated with their TPC content. In a study by Suárez et al. (2010), acetonic (70%) and methanolic (80%) extracts of AP exhibited considerable antioxidant properties. In addition, a 1.4 times more DPPH radical scavenging activity was observed in acetonic extract compared to the methanolic extract (Bai et al. 2013). Furthermore, polyphenols isolated from AP showed 2–3 times higher DPPH radical scavenging activity than that of an equal concentration of vitamins C or E (Lu and Foo 2000). The results, therefore, reinforce the potential of AP as a cheap and readily available antioxidant source.

## Conclusion

The kinetics of solid–liquid extraction of polyphenolic compounds from AP showed that the solvent type and the temperature have influence on TPC extraction efficiency. 65 ACE showed high efficiency in recovery of TPC. Furthermore, the study showed that high temperatures were not necessary for enhanced TPC extraction when the 65 ACE was used compared to when the solvents of 100 WA and 50 ETH were used. As expected, the DPPH of the TPC via the 65 ACE solvent exceeded the DPPH of the TPC via the 100 WA and 50 ETH solvents, in accordance with the TPC concentrations recorded. Crucially, the lower temperature requirement when 65 ACE is used may be translated to lower energy requirement and indeed improved economics in large-scale processes. The information presented in the study may also enhance the future designing and optimizing extraction processes and reduction of scaling-up costs.

### Supplementary Information

**Additional file 1.** Statistical significance of process variables of temperature and time on total polyphenol (TPC) extraction.

## Data Availability

All the needed data are provided in the manuscript.

## Abbreviations

TPC: Total phenolic compounds
AP: Apple pomace
GAE: Gallic acid equivalent
GHG: Greenhouse gas
DPPH: 2,2-Diphenyl-1 picrylhydrazyl
WAP: Waste apple pomace
100 WA: Water-only
50 ETH: 50% Ethanol–50% water v/v
65 ACE: 65% Acetone–35% water v/v
