# Supplementary material for: Kinetic modelling of the solid–liquid extraction process of polyphenolic compounds from apple pomace: influence of solvent composition and temperature
Source: Bioresour Bioprocess. 2021 Nov 24;8(1):114. doi: 10.1186/s40643-021-00465-4 (PMC10991919; doi:10.1186/s40643-021-00465-4)
Supplement: Supplementary file 1 — Additional file 1. Statistical significance of process variables of temperature and time on total polyphenol (TPC) extraction. [file 40643_2021_465_MOESM1_ESM.docx]

**Supplementary information**

**Kinetic modelling of the solid-liquid extraction process of polyphenolic compounds from apple pomace: Influence of solvent composition and temperature**

Parinaz Hobbi^a^, Oseweuba Valentine Okoro^a^, Christine Delporte^b^, Houman Alimoradi^c^, Daria Podstawczyk^d^, Lei Nie^e^, Katrien V. Bernaerts^f^, Amin Shavandi*^a^

^a^ Université libre de Bruxelles (ULB), École polytechnique de Bruxelles - BioMatter unit, Avenue F.D. Roosevelt, 50 - CP 165/61, 1050 Brussels, Belgium

*^b^*Laboratory of Pathophysiological and Nutritional Biochemistry, Université Libre de Bruxelles, Belgium

^c^ School of Biomedical Sciences, University of Otago, Dunedin, New Zealand

^d^ Department of Process Engineering and Technology of Polymer and Carbon Materials, Faculty of Chemistry, Wroclaw University of Science and Technology, Norwida 4/6, 50-373 Wroclaw, Poland

^e^College of Life Sciences, Xinyang Normal University (XYNU), Xinyang 464000, China

^f^ Maastricht University, Faculty of Science and Engineering, Brightlands Chemelot Campus, Aachen-Maastricht Institute for Biobased Materials (AMIBM), Urmonderbaan 22, 6167 RD Geleen, the Netherlands

**STATISTICAL SIGNIFICANCE OF PROCESS VARIABLES OF TEMPERATURE AND TIME ON TOTAL POLYPHENOL (TPC) EXTRACTION**

**CASE 1: water solvent**

Table S1: Analysis of variance of the experimental data for total polyphenolic (TPC) extraction using water solvent

| Source | DF | Adj SS | Adj MS | F-Value | P-Value |
| --- | --- | --- | --- | --- | --- |
| Time (min) | 5 | 120.721 | 24.1442 | 28.97 | 0.000 |
| Temperature (º C) | 2 | 15.834 | 7.9171 | 9.50 | 0.000 |
| Time*Temperature (min. º C) | 10 | 4.597 | 0.4597 | 0.55 | 0.841 |
| Error | 36 | 30.000 | 0.8333 |  |  |
| Total | 53 | 171.152 |  |  |  |

Tables S1 shows that while the parameters of time and temperature are statically significant (i.e. P value < 0.05), their interaction is not significant (P value > 0.05).

Based on the experimental data, the regression equation showing the dependence of TPC on different temperatures and time is determined as follows;

| TPC | = | 3.226 - 0.724 Temperature_40 + 0.577 Temperature_60 + 0.147 Temperature_85 - 3.226 Time_0 - 0.076 Time_5 + 0.474 Time_10 + 0.674 Time_15 + 1.091 Time_25 + 1.064 Time_30 + 0.724 Temperature*Time_40 0 - 0.146 Temperature*Time_40 5 - 0.306 Temperature*Time_40 10 - 0.106 Temperature*Time_40 15 + 0.068 Temperature*Time_40 25 - 0.236 Temperature*Time_40 30 - 0.577 Temperature*Time_60 0 + 0.423 Temperature*Time_60 5 + 0.013 Temperature*Time_60 10 - 0.117 Temperature*Time_60 15 + 0.036 Temperature*Time_60 25 + 0.223 Temperature*Time_60 30 - 0.147 Temperature*Time_85 0 - 0.277 Temperature*Time_85 5 + 0.293 Temperature*Time_85 10 + 0.223 Temperature*Time_85 15 - 0.104 Temperature*Time_85 25 + 0.013 Temperature*Time_85 30 |
| --- | --- | --- |

The associated correlation coefficient is determined to be 0.8247, highlighting model sufficiency in predicting dependence of TPC on process parameters of temperature and time .

**CASE 2: 50 v/v % ethanol + water solvent**

Table S2: Analysis of variance of the experimental data for total polyphenolic (TPC) extraction using 50 v/v % ethanol solvent

| Source | DF | Adj SS | Adj MS | F-Value | P-Value |
| --- | --- | --- | --- | --- | --- |
| Temperature | 2 | 27.311 | 13.656 | 17.07 | 0.000 |
| Time | 4 | 416.266 | 104.066 | 130.08 | 0.000 |
| Temperature*Time | 8 | 6.916 | 0.865 | 1.08 | 0.403 |
| Error | 30 | 24.000 | 0.800 |  |  |
| Total | 44 | 474.493 |  |  |  |

Table S2 shows that while the parameters of time and temperature are statically significant (i.e. P value < 0.05), their interaction is not significant (P value > 0.05).

Based on the experimental data, the regression equation showing the dependence of TPC on different temperatures and time is determined as follows;

| TPC | = | 6.071 - 0.945 Temperature_20 - 0.019 Temperature_40 + 0.963 Temperature_60 - 6.071 Time_0 + 1.196 Time_5 + 1.489 Time_10 + 1.593 Time_15 + 1.793 Time_25 + 0.945 Temperature*Time_20 0 - 0.302 Temperature*Time_20 5 - 0.235 Temperature*Time_20 10 - 0.259 Temperature*Time_20 15 - 0.149 Temperature*Time_20 25 + 0.019 Temperature*Time_40 0 - 0.008 Temperature*Time_40 5 + 0.039 Temperature*Time_40 10 + 0.035 Temperature*Time_40 15 - 0.085 Temperature*Time_40 25 - 0.963 Temperature*Time_60 0 + 0.310 Temperature*Time_60 5 + 0.197 Temperature*Time_60 10 + 0.223 Temperature*Time_60 15 + 0.233 Temperature*Time_60 25 |
| --- | --- | --- |

The associated correlation coefficient is determined to be 0.9494, highlighting model sufficiency in predicting dependence of TPC on process parameters of temperature and time .

**CASE 3: 65 v/v % acetone + water solvent**

Table S3: Analysis of variance of the experimental data for total polyphenolic (TPC) extraction using 65 v/v% acetone solvent

| Source | DF | Adj SS | Adj MS | F-Value | P-Value |
| --- | --- | --- | --- | --- | --- |
| Temperature | 2 | 16.396 | 8.198 | 9.84 | 0.000 |
| Time | 5 | 603.159 | 120.632 | 144.76 | 0.000 |
| Temperature*Time | 10 | 4.538 | 0.454 | 0.54 | 0.847 |
| Error | 36 | 30.000 | 0.833 |  |  |
| Total | 53 | 654.092 |  |  |  |

Table S3 shows that while the parameters of time and temperature are statically significant (i.e. P value < 0.05), their interaction is not significant (P value > 0.05).

Based on the experimental data, the regression equation showing the dependence of TPC on different temperatures and times is determined as follows;

| TPC | = | 7.359 - 0.431 Temperature_20 - 0.347 Temperature_40 + 0.778 Temperature_60 - 7.359 Time_0 + 0.618 Time_5 + 1.051 Time_10 + 1.378 Time_15 + 2.458 Time_30 + 1.854 Time_40 + 0.431 Temperature*Time_20 0 - 0.056 Temperature*Time_20 5 + 0.091 Temperature*Time_20 10 - 0.036 Temperature*Time_20 15 - 0.406 Temperature*Time_20 30 - 0.023 Temperature*Time_20 40 + 0.347 Temperature*Time_40 0 - 0.039 Temperature*Time_40 5 + 0.047 Temperature*Time_40 10 - 0.019 Temperature*Time_40 15 - 0.099 Temperature*Time_40 30 - 0.236 Temperature*Time_40 40 - 0.778 Temperature*Time_60 0 + 0.096 Temperature*Time_60 5 - 0.138 Temperature*Time_60 10 + 0.056 Temperature*Time_60 15 + 0.506 Temperature*Time_60 30 + 0.259 Temperature*Time_60 40 |
| --- | --- | --- |

The associated correlation coefficient is determined to be 0.9541, highlighting model sufficiency in predicting dependence of TPC on process parameters of temperature and time.
